# Experimental and theoretical insights into the effects of pH on catalysis of bond-cleavage by the lignin peroxidase isozyme H8 from *Phanerochaete chrysosporium*

**DOI:** 10.1186/s13068-021-01953-7

**Published:** 2021-04-29

**Authors:** Le Thanh Mai Pham, Kai Deng, Trent R. Northen, Steven W. Singer, Paul D. Adams, Blake A. Simmons, Kenneth L. Sale

**Affiliations:** 1 Joint BioEnergy Institute, Emeryville, CA 94608 USA; 2 Sandia National Laboratories, Livermore, CA 94550 USA; 3 Lawrence Berkeley National Laboratory, Berkeley, CA 94720 USA; 4 University of California, Berkeley, CA 94720 USA

**Keywords:** *Phanerochaete chrysosporium*, Lignin peroxidase, Lignin degradation, Ab initio molecular dynamic simulations, Quantum calculation

## Abstract

**Background:**

Lignin peroxidases catalyze a variety of reactions, resulting in cleavage of both β-O-4′ ether bonds and C–C bonds in lignin, both of which are essential for depolymerizing lignin into fragments amendable to biological or chemical upgrading to valuable products. Studies of the specificity of lignin peroxidases to catalyze these various reactions and the role reaction conditions such as pH play have been limited by the lack of assays that allow quantification of specific bond-breaking events. The subsequent theoretical understanding of the underlying mechanisms by which pH modulates the activity of lignin peroxidases remains nascent. Here, we report on combined experimental and theoretical studies of the effect of pH on the enzyme-catalyzed cleavage of β-O-4′ ether bonds and of C–C bonds by a lignin peroxidase isozyme H8 from *Phanerochaete chrysosporium* and an acid stabilized variant of the same enzyme.

**Results:**

Using a nanostructure initiator mass spectrometry assay that provides quantification of bond breaking in a phenolic model lignin dimer we found that catalysis of degradation of the dimer to products by an acid-stabilized variant of lignin peroxidase isozyme H8 increased from 38.4% at pH 5 to 92.5% at pH 2.6. At pH 2.6, the observed product distribution resulted from 65.5% β-O-4′ ether bond cleavage, 27.0% C_α_-C_1_ carbon bond cleavage, and 3.6% C_α_-oxidation as by-product. Using ab initio molecular dynamic simulations and climbing-image Nudge Elastic Band based transition state searches, we suggest the effect of lower pH is via protonation of aliphatic hydroxyl groups under which extremely acidic conditions resulted in lower energetic barriers for bond-cleavages, particularly β-O-4′ bonds.

**Conclusion:**

These coupled experimental results and theoretical explanations suggest pH is a key driving force for selective and efficient lignin peroxidase isozyme H8 catalyzed depolymerization of the phenolic lignin dimer and further suggest that engineering of lignin peroxidase isozyme H8 and other enzymes involved in lignin depolymerization should include targeting stability at low pH.

**Supplementary Information:**

The online version contains supplementary material available at 10.1186/s13068-021-01953-7.

## Background

Lignocellulosic biomass, which is composed mainly of cellulose, hemicellulose, and lignin, is the most abundant source of renewable carbon on Earth [1], and the aromatic biopolymer lignin is the most abundant source of renewable aromatics [2]. Currently, most lignin is either stockpiled or burned to generate power, but techno-economic models of lignocellulosic biorefineries show that diverting lignin from on-site combustion saves costs [3] and converting lignin to value-added biofuels and bioproducts greatly improves the economics of biorefineries and reduces greenhouse gas (GHG) emissions [4]. As a first step in the process of converting lignin to valuable products, depolymerization of lignin to biologically available intermediates is critical. Unfortunately, efficient enzymatic depolymerization of lignin to biologically available intermediates at high yields has been impeded by a limited fundamental understanding of how lignin-degrading enzymes function and, more importantly, how we might control the activity of these enzymes to produce the most biologically useful lignin fragments.

In nature, fungi and bacteria produce an array of enzymes, including laccases, lignin peroxidases (LiP), versatile peroxidases (VP), and manganese peroxidases (MnP), that are secreted into the environment and catalyze depolymerization of lignin [5, 6]. In general, numerous physio-chemical factors such as pH, ionic strength, enzyme concentration, and redox potentials are potential driving forces for depolymerization of lignin but in some cases may also drive the re-polymerization of the lignin fragments produced during depolymerization. Some microorganisms involved in biomass degradation actively modify the pH of their environment via secretion of acids and bases, and fungi produce organic acids, resulting in significant acidification of conditions in their microenvironment, which is thought to play many key roles in nature [7–9]. Notably, two *Basidiomycota* known to degrade lignin*, Phanerochaete chrysosporium*, and *Trametes menziesii,* have been shown to acidify their environment to pH 2 or lower, implicating the pH of the fungal environment as a key driving force for the biological breakdown of biomass and specifically for depolymerization of lignin catalyzed by LiP, MnP, VP and laccases [10].

The fungi *P. chrysosporium* and *T. menziesii* employ extracellular peroxidases including LiP, VP, and MnP to catalyze bond cleavage in lignin [11–13]. One particular well-studied enzyme is the lignin peroxidase isozyme H8 (LiPH8) from *P. chrysosporium,* which harbors a surface exposed catalytic Trp171 amino acid known to play a vital role in the oxidation of high-redox-potential mediators such as veratryl alcohol (VA) and nonphenolic lignin derivatives [14]. The VA cation radical has been proposed as a radical mediator to oxidize polymeric substrates with which LiP presumably cannot interact directly [15–17]. The mediator radical withdraws an electron from lignin resulting in a cationic radical intermediate and ultimately producing a wide variety of ligninolytic breakdown products through non-specific bond-cleavage occurrences [18, 19].

LiPs have been shown to have higher activity toward oxidation of compounds such as 2,2′-azino-bis(3-ethylbenzothiazoline-6-sulfonic acid (ABTS) [20], VA [21–25] and the azo dye reactive black 5 [20] at lower pH.

Stopped-flow techniques have been used to investigate the kinetics of the reaction of LiP compounds I and II (LiPI and LiPII) with VA, and the formation of LiPI and LiPII in the presence of VA was strongly dependent on pH [25–27]. In addition, LiPH8 from *P. chrysosporium* has been shown to catalyze oxidative cleavage of both β-O-4′ ether and C–C bonds in aryl ether dimers [28] and to catalyze breaking of β-O-4′ ether, C–C, and C-H bonds in trimeric lignin model compounds [29]. Combined, these studies suggest LiPH8 should catalyze β-O-4′ ether and C–C at higher rates as pH is reduced, but studies aimed at understanding bond cleavage as a function of pH and describing potential pH-dependent mechanisms have not been performed.

To partially address this lack of knowledge for LiP, the work presented here is focused on developing a fundamental understanding of LiPH8– catalyzed depolymerization of a phenolic lignin-like dimer compound as a function of pH. We present detailed studies of the effect of pH on the enzyme-catalyzed cleavage of β-O-4′ ether bonds and C–C bonds by LiPH8 from *P. chrysosporium* using a recently developed assay based on model lignin-like dimers and nanostructure initiator mass spectrometry (NIMS) [30]. Using the NIMS assay, we quantified dimer conversion and the distribution of products formed by breaking C–C and β-O-4′ ether bonds upon incubation of a phenolic lignin-like model compound with LiPH8. We show the distribution of products is pH-dependent, suggesting pH adjustments can be used to control LiPH8 catalyzed breaking of β-O-4′ ether bonds and C–C bonds and production of lignin-derived compounds. We also report on our use of quantum calculations and ab initio molecular dynamic (AIMD) simulations to generate a fundamental understanding of how pH assist bond cleavage and show the roles of low pH are to i) provide a thermodynamically favorable condition for the formation of a cation radical intermediate required for energy-favorable degradation of lignin, ii) pH-assisted formation of protonated cationic radical intermediate at both phenolic and aliphatic hydroxyl groups, resulting in higher cleavage frequencies for various bond types in lignin, especially β-O-4′ ether bonds.

## Results and discussion

### pH-controlled catalysis of bond cleavage by the lignin peroxidase from *P. chrysosporium*

**Fig. 1:**
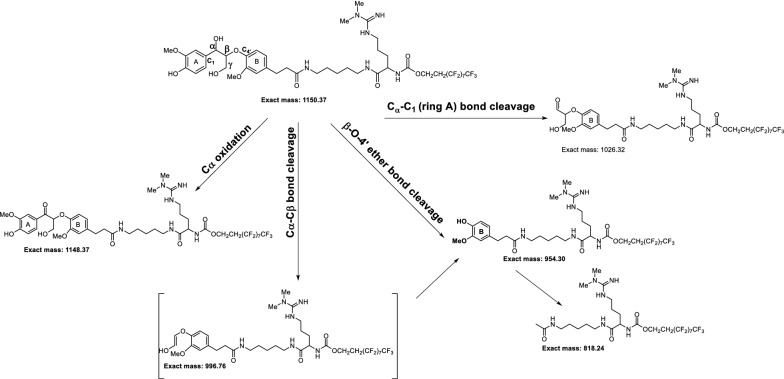
Representative products from bond cleavage events of the GGE-NIMS dimer used to assay LiPH8 from *Phanerochaete chrysosporium*

**Fig. 2:**
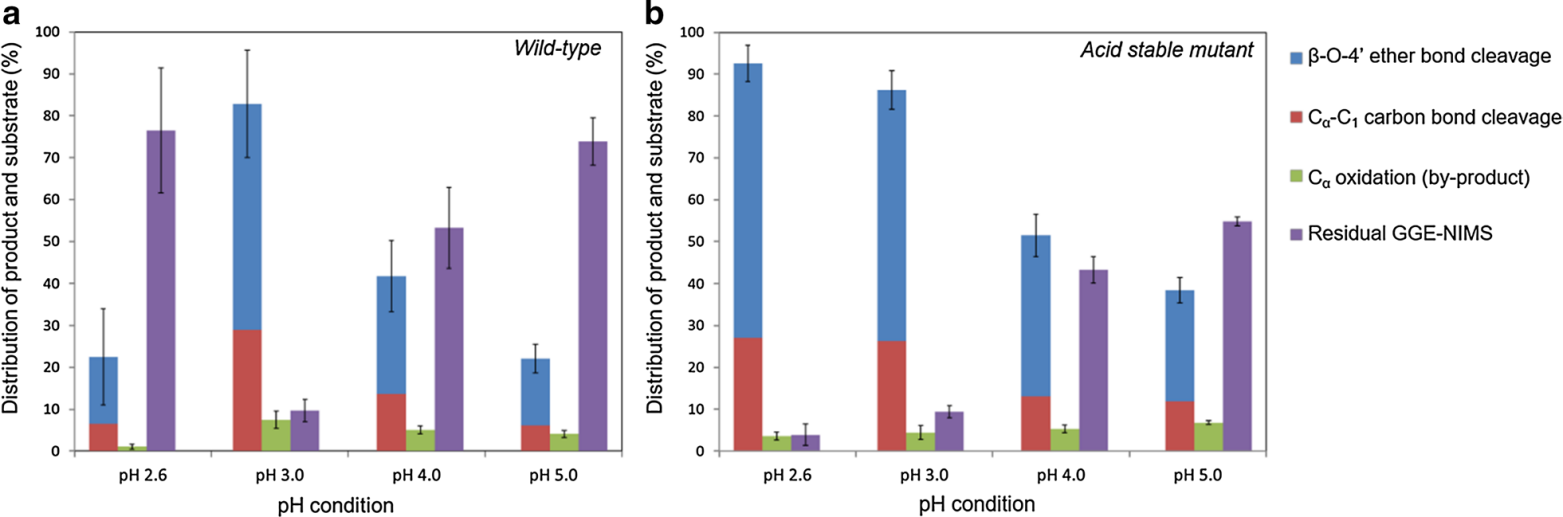
Product distribution from bond cleavage of fluorous-tagged GGE by wildtype lignin peroxidase (**a**) and acid-stabilized mutant of the same peroxidase (**b**). The reaction contained 1 mM of NIMS-tagged phenolic lignin dimer, 5 µM of LiPH8 enzyme, 20 mM of veratryl alcohol as mediator was performed in sodium acetate buffer pH 2. 6–5.0. The reaction was started by adding H_2_O_2_ fed at a rate of 250 uM every 30 min up to 3 h. Error bars are the standard deviation for three replicates, and the first column at each pH condition in Fig. 1 is the mean and standard deviation of the products from both β-O-4′ ether and C_α_-C_1_ bond cleavage combined. The means and standard deviations for β-O-4′ bond cleavage were 65.5 ± 0.8 (pH 2.6), 60.0 ± 1.3 (pH 3.0), 38.4 ± 1.0 (pH 4.0) and 26.5 ± 0.4 (pH 5.0). The means and standard deviations for C_α_-C_1_ bond cleavage were 27.0 ± 4.3 (pH 2.6), 26.24 ± 4.5 (pH 3.0), 13.0 ± 5.0 (pH 4.0 and 11.9 ± 3.0 (pH 5.0)

Lignin is an unusual biopolymer because of its structural heterogeneity, which has limited detailed studies of lignin-degrading enzymes in terms of their ability to catalyze the breaking of specific bond types. The most predominant linkage between phenylpropane units in both softwood and hardwood lignin is the β-O-4′ ether bond, comprising 45 – 60% [31, 32] of the bonds in lignin. It was found that LiPH8 directly interacts with the synthesized lignin macromolecules, which was supported by kinetic analysis of its binding affinity [33]. However, quantitative detection of phenolic products has not been reported for in vitro depolymerization of lignin by LiPH8. It is hypothesized that repolymerization of degraded lignin fragments spontaneously occurs and presents a barrier to in vitro depolymerization as well as to the detection of fragments produced and subsequent analysis of bond cleavage specificity. Thus, devising schemes to control depolymerization of lignin to produce defined intermediates requires a detailed understanding of reaction mechanisms and conditions, which are not well understood for this enzyme and, in general, for enzymes shown to be involved in lignin depolymerization. Herein, we study catalysis of bond cleavage events in a phenolic lignin dimer by quantitative analysis of product formation during LiPH8-catalyzed degradation of a GGE model compound (GGE-NIMS compound) using nanostructure-initiator mass spectrometry (GGE-NIMS dimer structure shown in Fig. 1 [30]. The enzyme assay was experimentally measured at four pH levels between 2.6 and 5.0, which are levels previously determined to result in the high activity of VA oxidation for wild-type LiPH8 and a low pH stabilized variant of it [23]. Products from the oxidation of the GGE-NIMS dimer through a one-electron reaction step were quantified using the NIMS assay and resulted from catalysis of four main reactions: C_α_-oxidation, C_α_-C_β_ bond cleavage, β-O-4 ether bond cleavage, and C_α_-C_1_ carbon bond (ring A) cleavage (Fig. 1). The data presented in Fig. 2a show that wildtype LiPH8 exhibited its highest catalytic capability at pH 3.0, with greater than 90% of the GGE-NIMS dimer being converted to products. At pH  <  3.0 (pH = 2.6), wildtype LiPH8 lignin peroxidase was essentially inactivated, resulting in more than 75% of the GGE-NIMS substrate being unmodified (Fig. 2a). At all pH levels, β-O-4′ ether bond cleavage released phenolic product (m/z 954.3, Fig. 1), which was the dominate catalytic event, followed by C_α_-C_1_ carbon bond cleavage and C_α_ oxidation.

To further investigate the effects of acidic conditions on bond cleavage frequency and specificity, a previously reported acid-stabilized variant of LiPH8 was assayed over pH ranging from 2.6 to 5.0 [23]. The triple mutant A55R-N156E‑H239E LiPH8 with 2 additional salt-bridges on the solvent-exposed regions showed excellent stability and oxidation activity under extremely acidic conditions down to pH 2.6 [23]. The stabilized mutant showed higher activity levels at all three pH levels tested and had the highest activity at pH 2.6 (Fig. 2b). Comparison of the residual GGE-NIMS substrate (Fig. 2b, purple bars) shows a clear upward trend of increased conversion of lignin dimer, converting 96.1% and 45.3% of the dimer at pH 2.6 and pH 5.0, respectively. The higher conversion of substrate resulted in more products from various bond cleavage events under the catalysis of the acid-stable mutant. For instance, β-O-4 ether bond cleavage was elevated from 26.5% to 65.5%, and products from C_α_-C_1_ carbon bond cleavage were also increased from 11.9% to 27.0% when pH decreased from 5.0 to 2.6.

While very acidic pH conditions drove the reaction to be more complete, the distributions of products produced from β-O-4′ ether bond and C_α_-C_1_ carbon bond cleavage at any of the pH levels studied were very similar. The ratio of product from β-O-4′ ether bond cleavage to product from C_α_-C_1_ carbon bond was distributed over a narrow range between 2.7 and 2.2 when acidifying pH from 5.0 to 2.6, while C_α_ oxidation events decreased from 6.8% to 3.6% between pH 5.0 and 2.6, respectively. These results suggested pH affects overall bond-cleavage frequency but does not determine product fragmentation pathways. Further study with various lignin dimer types may be needed to better understand the structural effect on product fragmentation pathways.

Exactly how in nature fungi alter the pH of their environment is not exactly know, but white-rot basidiomycetes have been shown to secret organic acids into their microenvironment, which could assist extracellular ligninase catalyzed lignin degradation [10]. This is in agreement with other studies showing that LiP and VP catalyze VA and non-phenolic lignin dimer oxidation with a pH optimum of 3.0 [20, 24, 25, 34–36]. It has been suggested through experimental study and quantum mechanics/molecular mechanics calculations that the redox potential of heme peroxidases increases at low pH, providing LiP and VP the capability to oxidize the recalcitrant lignin polymer at low pH [25, 37, 38]. Another explanation put forth is that the acidic residues surrounding the surface Trp171 that interacts with either lignin or mediators may be involved in the composition of an acidic environment, which acts to stabilize radical cation intermediates of small phenolic compounds [14]. Herein, we propose a pH-dependent mechanism in which the lignin dimer is protonated to different degrees at different pH values and study these proposed mechanisms using quantum calculation and AIMD simulation study.

### Low pH conditions drive reaction equilibrium toward the favorable formation of the active cationic radical intermediate

**Fig. 3:**
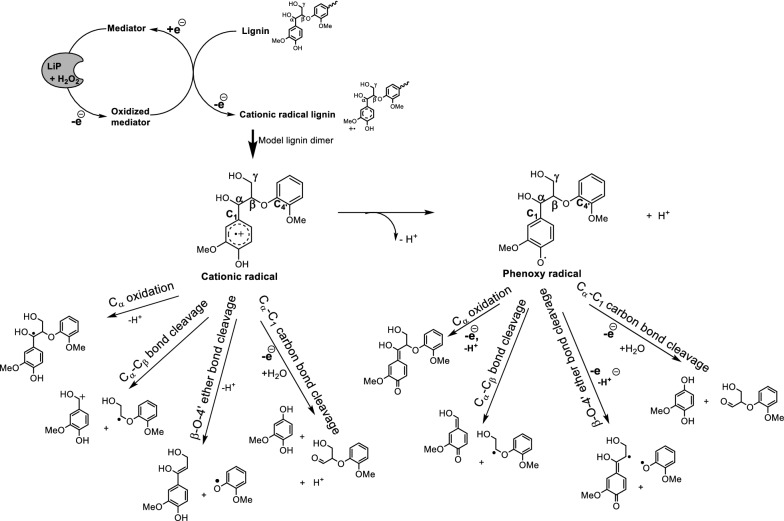
The proposed scheme of LiPH8/H_2_O_2_ catalyzed depolymerization and mechanisms and bond cleavage pathways from phenolic lignin dimer via either a cationic radical (left figure) or a phenoxy radical intermediate (right figure)

We proposed that bond cleavage in the GGE dimer occurs via either cationic or phenoxy radical intermediates and investigated the relative free energies for each pathway. The cationic radical intermediate formed from LiPH8/H_2_O_2_–catalyzed 1-electron oxidation of GGE dimer is capable of undergoing a variety of reactions such as side-chain oxidation, C–C bond, and β-O-4′ ether bond cleavage. The intermediates were predicted from a heterolytic bond cleavage reaction mechanism when the first step 1-electron oxidation takes place at lower redox potential–Ring A (Fig. 3). The deprotonation of the short-lived cationic radical resulted in the formation of the phenoxy radical which sequentially cleaved into fragments (Fig. 3).

**Fig. 4:**
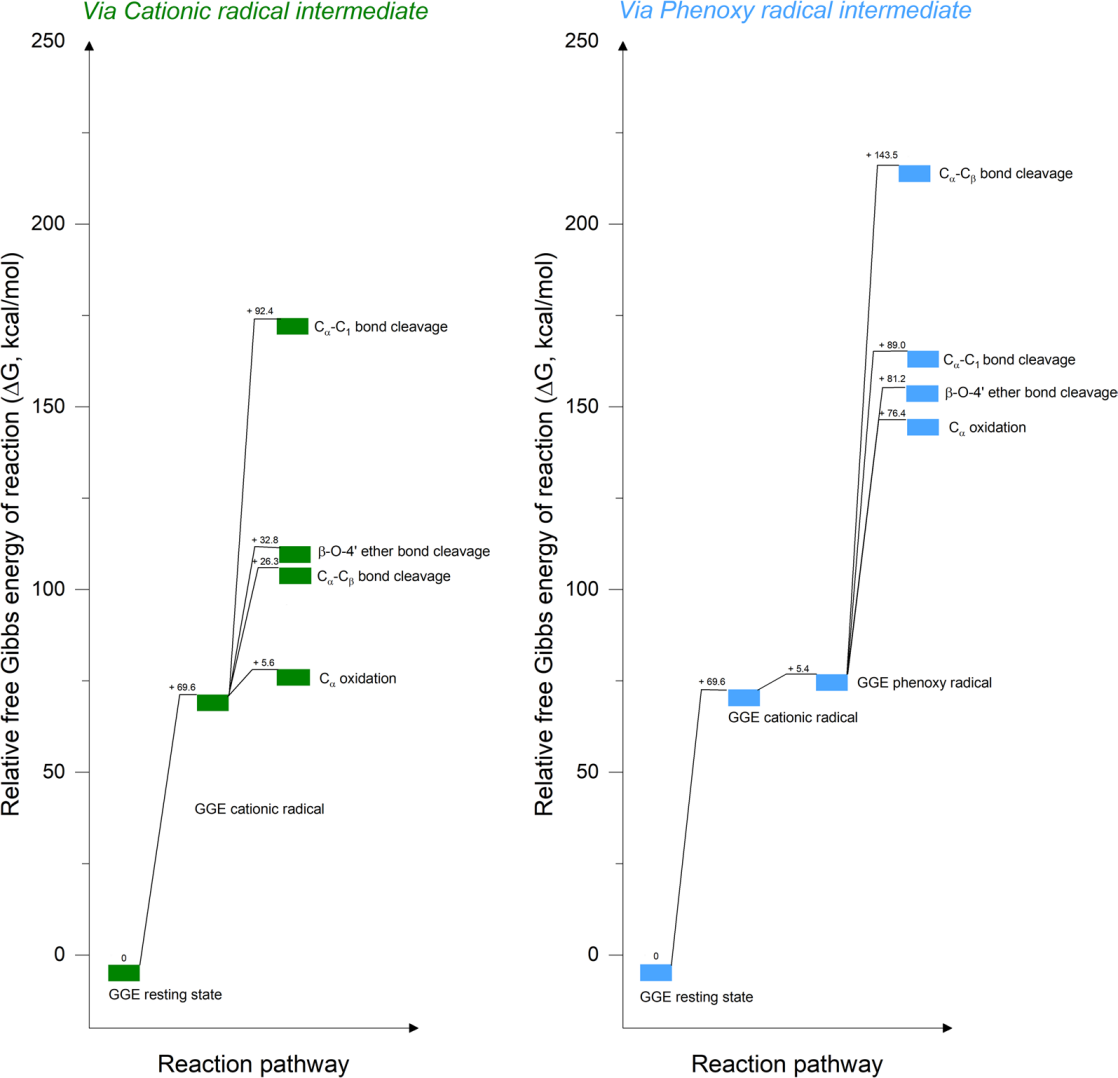
Relative Gibbs free energy required for breaking various bond types in lignin via the cationic radical intermediate (left) and the phenoxy radical intermediate (right). Gibbs free energy was normalized to reactants at each reaction step

The relative calculated Gibbs free energy for the occurrence of bond cleavage via either the cationic radical intermediate or the phenoxy radical intermediate indicated that much high energy is required for bond cleavage to occur from the phenoxy radical intermediate compared to the free energy required when starting from the cationic radical intermediate (Fig. 4). For instance, the calculated Gibbs free energy required for β-O-4′ ether bond cleavage from the cationic radical is 32.8 kcal/mol versus 81.2 kcal/mol from the phenoxy radical intermediate. These results indicate that stabilizing the radical cation and decreasing the deprotonation rate will drive bond-cleavage occurrence via the cationic radical intermediate, suggesting an approach for controlling lignin depolymerization rates. The position of the equilibrium will depend on the countercation and solvent, and herein we suggest an approach of H^+^ concentration-controlled protonation/deprotonation equilibrium between cationic radicals and phenoxy radicals. Simply, according to LeChâtelier’s principle, the addition of H^+^ ions (as in a low pH solution) drives the equilibrium to the left and the protonated cationic radical predominates. This finding is consistent with the natural modulation of the significant acidic condition in the microenvironment around fungi cells which was previously reported by Liaud et al. [10].

### Protonation of hydroxyl group under acidic conditions is a key step in bond-cleavage pathways

**Fig. 5:**
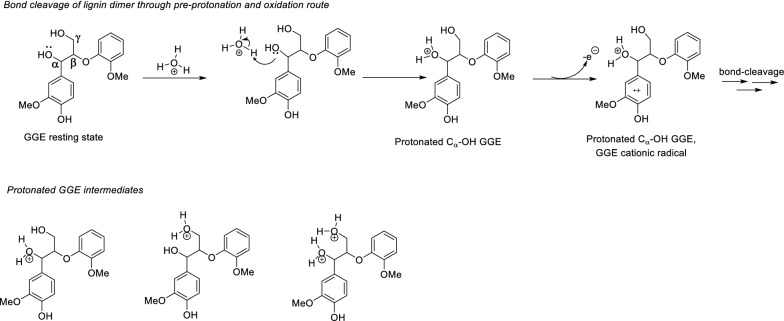
Proposed routes to bond-cleavage through a pre-protonation mechanism and an oxidation route and their respective protonated intermediates

Regarding lignin’s functional groups, aliphatic hydroxyl and phenolic hydroxyl groups have been shown to significantly affect the reactivity of lignin by nucleophilic aromatic substitution reactions including amination, nitration, and esterification [39–41]. Many studies have reported on the effects of these functional groups on depolymerization mechanisms. In reductive depolymerization, Wang and co-workers showed that the aliphatic alcohol moieties (C_α_H − OH) in lignin itself can act as the hydrogen donor [42]. In oxidative depolymerization, under acidic conditions, stabilization of aliphatic hydroxyl groups by formic acid and formaldehyde resulted in increased product yield of monomers [43, 44]. Herein, we hypothesized that under more acidic conditions formation of protonated hydroxyl groups drives the reaction to further completion by lowering bond dissociation energies. Under acidic conditions, hydronium cations react with aliphatic OH groups (C_α_-OH, C_γ_-OH), and phenolic-OH groups to form protonated C_α_-OH GGE, protonated C_γ_-OH GGE and protonated phenolic-OH GGE intermediates, respectively. Gibbs free energies were calculated from single-point energies at the B3LYP/6-311G**/SMD_water_ level of theory for the formation of the protonated hydroxyl GGE cationic radicals formed through either a pre-protonation-oxidation route (Additional file 1: Figure S1) or a pre-oxidation protonation route (Additional file 1: Figure S2). The calculated Gibbs free energies suggest that oxidation of GGE and subsequent bond cleavage via the pre-protonation route required much lower energies compared with the pre-oxidation protonation route. Specifically, these data strongly suggest that the formation of protonated C_α_-OH/C_γ_-OH GGE intermediates is more favorable than the formation of a protonated phenolic-OH GGE intermediate (Additional file 1: Figure S1). The protonated C_α_-OH GGE and protonated C_γ_-OH GGE can spontaneously form under acidic conditions, while the formation of protonated phenolic-OH GGE intermediates requires either more energy or strong acids. Three kinds of protonated aliphatic-OH GGE intermediates can be formed through the pre-protonation oxidation route, and under the catalysis of the acid-stabilized LiPH8 mutant protonated hydroxyl GGE cationic radicals are formed and further cleaved into product fragments (Fig. 5).

For each protonated hydroxyl GGE cationic radical intermediate, we carried out 5 ps of AIMD simulations and counted bond-breaking events to determine the frequency of each type of bond-breaking event as a function of protonation of GGE hydroxyl groups at different positions on GGE (Fig. 5). In the absence of protonation, only β-O-4′ ether bond cleavage was observed for the non-protonated GGE cationic radical with an 11.42% bond cleavage efficiency. As hypothesized above, protonated GGE cationic radical intermediates are formed when the solution is acidified to lower pH and a higher frequency of β-O-4′ ether bond cleavages was observed for protonated GGE intermediates at C_α_-OH or C_γ_-OH position. The highest β-O-4′ ether bond cleavage frequency (33.66%) was observed for the GGE cationic radical intermediate in which our calculations show protonation would have occurred at both the C_α_-OH and C_γ_-OH groups. This trend in β-O-4′ ether bond cleavage frequency is consistent with the experimental data reported in Fig. 2, which shows an increase in the β-O-4′ ether bond cleavage for the acid-stable LiPH8 as the pH drops from 5 to 2.6. The AIMD simulations also showed additional C_α_-C_1_ bond-breaking (3.86%) in the protonated hydroxyl GGE intermediates, and these were not observed for AIMD simulation of inert non-protonated OH GGE cationic radical intermediates. In comparison with the experiment, the content of the product from C_α_-C_1_ carbon bond increased from 11.9% to 27.0% when pH was shifted from 5.0 to 2.6 when using the acid-stable lignin peroxidase.

**Fig. 6:**
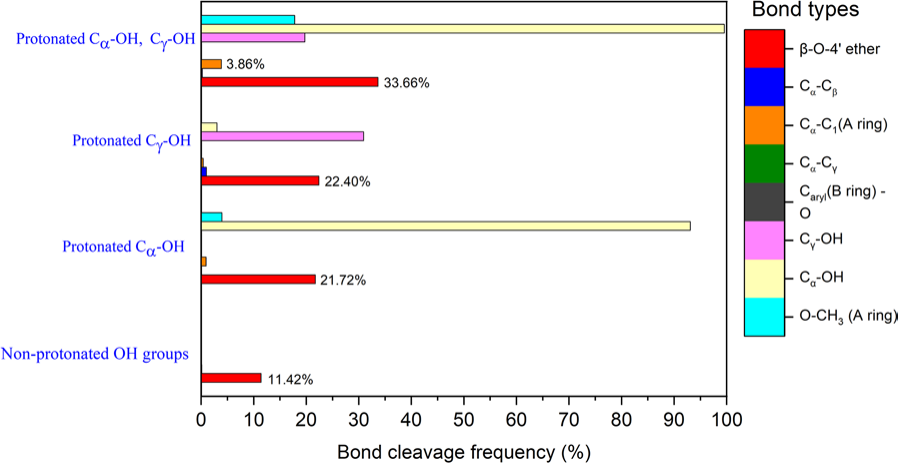
Bond-cleavage frequency computed by AIMD simulation of non-protonated and protonated OHs GGE cationic radical for different linkages

For C_α_-C_β_ bond cleavage, it is more energetically favorable than C_α_-C_1_ carbon bond cleavage; however, C_α_-C_β_ bond cleavage products were not detected experimentally (Figs. 2 and 4). The AIMD simulations also showed higher bond-cleavage frequency for C_α_-C_β_ bonds than for C_α_-C_1_ bonds (Fig. 6). This can be explained by noting the product formed from C_α_-C_β_ cleavage in Fig. 1 is not stable and would likely decompose rapidly to the structure obtained from β-O-4′ ether bond cleavage (Fig. 1). At the current state of the assay, we do not have a tool to quench reactions and trap these unstable products from C_α_-C_β_ bond cleavage; however, quantum calculations and AIMD simulation data provided an understanding of the existence of different pathways and interconversion of product intermediates.

**Fig. 7:**
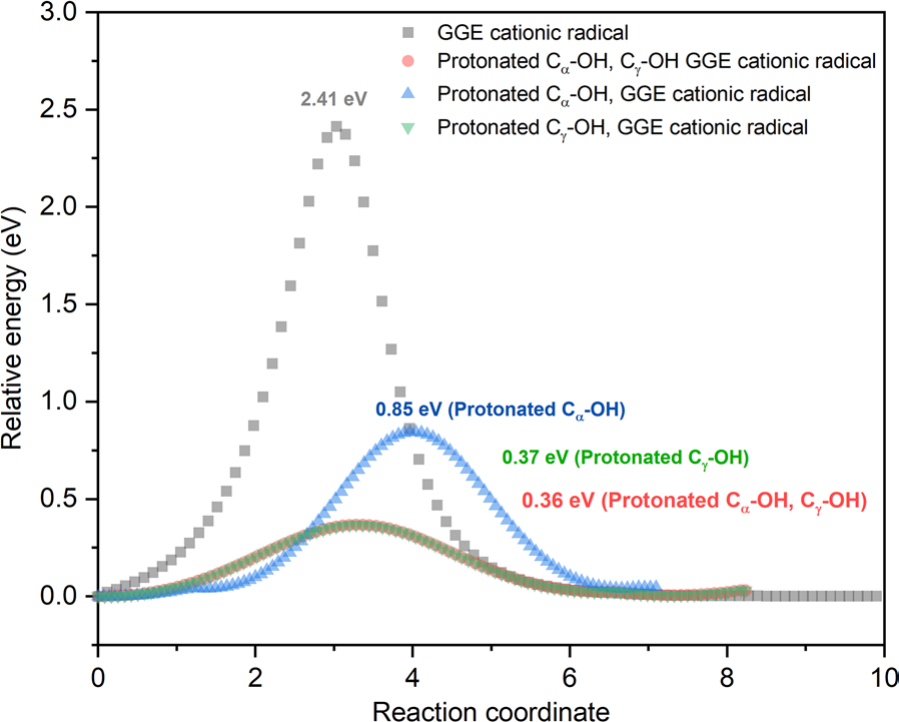
Reaction path computed with NEB for β-O-4′ ether bond cleavage from non-protonated and protonated hydroxyl GGE cationic radical intermediates

We also found that deprotonation at C_α_ and C_γ_ occurs in the AIMD simulations of protonated C_α_-OH/ C_γ_-OH GGE intermediates, suggesting it may play a role in catalysis of C–C bond and β-O-4′ ether bond breaking. To further investigate this, we computed the energetic barriers for the conversion of the GGE cationic radical intermediate to products via β-O-4 ether bond cleavage events observed in the AIMD trajectory analysis. C_α_-OH_2_ or C_γ_-OH_2_ bond cleavage was initiated and deprotonation of C_γ_-OH or C_β_-H was found in the reaction path of β-O-4′ ether bond cleavage (Additional file 1: Figure S3, S4). These structures were used as intermediates in NEB TS searches, which were computed using TeraChem at the unrestricted B3LYP/6-311G**/PCM_water_ level of theory (Fig. 7). Although the procedure of NEB calculation does not guarantee convergence to an exact transition state, it nonetheless provides the saddle points connecting two minima through pre-defined intermediates. It was found that the maximum relative energy for the transition state of non-protonated GGE cationic radicals was 2.41 eV. Protonation at either aliphatic C_α_-OH or C_γ_-OH resulted in a lower energy barrier for β-O-4′ ether bond cleavage occurrences. The lowest energy barrier of 0.36 eV was for the protonated intermediate at both C_α_-OH and C_γ_-OH position. A proposed mechanism for β-O-4′ ether bond cleavage from protonated C_α_-OH/ C_γ_-OH GGE cationic radical was suggested in Additional file 1: Figure S5, S6. In this proposed mechanism, an aliphatic hydroxyl C_α_-OH/ C_γ_-OH is protonated by H^+^ from the acid reagent, forming an alkyloxonium ion + OH_2_. This ion then, which is primed to acts as a good leaving group, leaves to form a carbocation at either the C_α_ or the C_γ_ position. The benzyl radical then acts as an electron-withdrawing group, which induces electron reorganization and C-O bond-cleavage. This simulation data matched with AIMD simulation where protonation at either C_α_-OH or C_γ_-OH resulted in higher cleavage frequencies for various bond types. Again, the combination of AIMD simulation and NEB-based TS searches matched our experimental data where protonated hydroxyl GGE intermediates were formed by acidifying reaction solution and sequentially resulted in a lower energy barrier for bond cleavages especially β-O-4′ ether, and higher conversion yield of dimer degradation.

#### Conclusion

We investigated the effects of pH on the activity of the lignin peroxidase isozyme H8 from *P. chrysosporium* using a novel nanostructure-initiator mass spectrometry assay for studying catalysis of bond breaking in a fluorous-tagged β-O-4′ phenolic lignin dimer model compound synthesized for use with this assay. Results from these assays showed increases in catalysis of β-O-4′ ether and C_α_-C_1_ carbon bond-breaking events and little change in oxidation at the C_α_ position as the reaction conditions became more acidic. To better understand how lower pH enhances bond-breaking reactions in the β-O-4′ lignin model compound we ran a series of computations to examine bond cleavage via either a cationic radical intermediate or a phenoxy radical intermediate. Our calculated relative Gibbs free energy for the occurrence of bond cleavage indicated that much high energy is required for bond cleavage to occur from the phenoxy radical intermediate compared to the Gibbs free energy required when starting from the cationic radical intermediate. Data from AIMD simulations and climbing-image NEB-based transition state searches suggested that under more acidic conditions formation of protonated hydroxyl groups drives the reaction to further completion by lowering energetic barriers for bond-cleavage, especially β-O-4′ bonds. This work generates a more robust fundamental understanding of how pH assists bond cleavage and provides insights into the optimal design of reaction conditions to improve the efficiency of lignin peroxidase-catalyzed phenolic lignin degradation.

## Materials and methods

### Materials

Hydrogen peroxide, hemin, oxidized glutathione, ampicillin, isopropyl-β-d-thiogalactopyranoside, guanidine hydrochloride, dibasic potassium phosphate, citric acid, trizma hydrochloride, and guaiacol used in this study were purchased from the Sigma Chemical Co., U.S and were used without any further purification.

Synthesis of phenolic β-O-4 ether model compound (3,3,4,4,5,5,6,6,7,7,8,8,9,9,10,10,10-heptadecafluorodecyl (19-(4-((1,3- dihydroxy-1-(4-hydroxy-3-methoxyphenyl)propan-2-yl)oxy)-3- methoxyphenyl)-3-imino-2-methyl-9,17-dioxo-2,4,10,16-tetraazanonadecan8-yl)carbamate) followed previously reported methods [30].

### Recombinant enzyme preparation

The gene coding for the wild-type lignin peroxidase LiPH8 (UniprotKB ID: P06181) and for the acid stabilized variant with three mutated residues (A55R/N156E‑H239E) was retrieved from a previously published report [23]. The expression in *E. coli*, refolding and purification procedures for both the wild-type enzyme and the pH stabilized enzyme were performed as previously reported [45].

### Enzyme reaction with flourous-tagged phenolic β-O-4 dimeric model compound

Enzyme reaction with NIMS-tagged phenolic lignin dimer (1 mM) was performed in sodium acetate buffer pH 2. 6–5.0 in the presence of 20 mM of veratryl alcohol as mediator. 5 µM enzyme was used in this reaction and the reaction was started by adding H_2_O_2_ fed at a rate of 250 uM every 30 min up to 3 h. The reported data are the mean and standard derivations for n = 3 replicates. The first column at each pH condition in Fig. 1 is the mean and standard of the products from both β-O-4′ ether and C_α_-C_1_ bond cleavage combined.

### Nanostructure‑initiator mass spectrometry

A protocol for analyzing fluorous-tagged guaiacylglycerol-β-guaiacyl ether (GGE) using NIMS and reaction products was performed as previously described [30]. Briefly, 0.2 µL of the quenched reaction sample was spotted onto the NIMS surface and removed after 30 s. The spotting and identification of sample spots in the spectrometer were manually drawn on the NIMS chip using a diamond-tip scribe. The modified matrix-assisted laser desorption (MALDI) plate containing chip was loaded into Bruker Ultraflextreme MALDI TOF/TOF mass analyzer. Signal intensities were identified for the ions of the products and ~ 5000 laser shots were collected for each sample.

### Simulation method

**Scheme 1:**
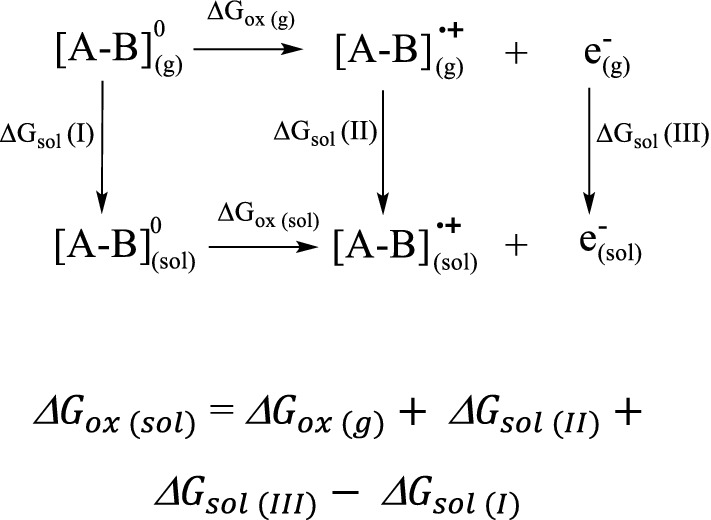
Born-Haber thermodynamic cycle for calculating the Gibbs free energy of Guaiacylglycerol-β-guaiacyl ether (GGE) dimer oxidation

The Gibbs free energy of oxidation of the GGE dimer into the cationic radical intermediate and bond dissociation for all bond types in GGE was defined as the standard-state Gibbs free energy change for the reaction at a specified temperature, here at 298 °K. Simulations were performed in the Gaussian 09 software package [46] with unrestricted density functional theory (DFT), Becke three-parameter exchange and Lee–Yang–Parr correlation (B3LYP) [47, 48] using the 6-311G** basis set [49, 50] and implicit solvation model based on density (SMD) [51]. The effect of solvent was modeled using the Born-Haber Cycle as shown in the following scheme 1:

Where ${G}_{ox (g)}$ is Gibbs free energy for oxidation in the gas phase at 298 °K, ${G}_{sol (I)}$ is the energy with solvent effect for the resting-state dimer; ${G}_{sol (II)}$ is the energy with solvent effect for the cationic radical dimer; ${G}_{sol (III)}$ is the energy with solvent effect for electron, which was obtained from the absolute protonation free energy of the solvated electron in water as − 35.5 kcal/mol using a reliable computational protocol of first-principles solvation-included electronic structure calculations [52].

AIMD simulations were carried out using the TeraChem quantum chemistry package (Petachem LLC, CA) [53–56]. The simulations were performed with unrestricted density functional theory (DFT) using the long-range corrected ωPBEh exchange–correlation functional [57] and the 6-31 g basis set. Simulations were run for 5 ps using a 1 fs time step. An unrestricted, radical solution, level-shifting, and a 1.0 eV and 0.0 eV shift were applied to α states and β states, respectively. TeraChem uses v6.0 of the NBO package for a full natural bond orbitals (NBO) analysis. Bond breaking events were defined as a separation distance between two atoms from fragments being longer than the corresponding bond length of initial model structure, and bond cleavage frequency was counted over 5000 AIMD time steps for the following linkages in the lignin dimer: C_α_-OH bond, C_α_-C_β_ bond, β-O-4′ ether bond and C_α_-C_1_ carbon bond.

The climbing-image Nudge Elastic Band (NEB) method was used to calculate the relative energy of the transition state through a reaction path defined with a set of images (initial, intermediate, and final images) taken from the analysis of AIMD simulation trajectories. The number of NEB images in the transition state search calculation was set to 100 and the min and max spring constants between NEB images were set to 0.005 and 0.05, respectively. The solvation model based on the conductor-like solvation model (COSMO) [58] was performed with a water dielectric of ε = 78.39.

## Supplementary Information

**Additional file 1: Figure S1.** Relative Gibbs free energy for the formation of protonated intermediates at phenolic OH (blue bar), C_α_-OH (green bar) and C_γ_-OH (orange bar) positions through a pre-protonation – oxidation route. Gibbs Free energy was normalized to reactants on each reaction step. **Figure S2.** Relative Gibbs free energy for the formation of protonated intermediates at phenolic OH (blue bar), C_α_-OH (green bar) and C_γ_-OH (orange bar) positions through a pre-oxidation – protonation route. Gibbs Free energy was normalized to reactants on each reaction step. **Figure S3.** Snapshots of intermediates from AIMD simulation of the protonated C_α_-OH, GGE cationic radical for β-O-4′ ether bond cleavage. **Figure S4.** Snapshots of intermediates from AIMD simulation of protonated C_γ_-OH, GGE cationic radical for β-O-4′ ether bond cleavage. **Figure S5.** Proposed mechanism for β-O-4′ ether bond cleavage from protonated C_α_-OH, GGE cationic radical. **Figure S6.** Proposed mechanism for β-O-4′ ether bond cleavage from protonated C_γ_-OH, GGE cationic radical.

## Data Availability

The datasets used and/or analyzed during the current study are available from the corresponding author on reasonable request. Protein sequence: UniprotKB ID P06181. *E. coli* strains containing the genes of interest are available from the. Joint BioEnergy Institutes strain archive. Strain JBx_137395: *E. coli* DH5α contains pET-21b( +) plasmid cloned with lignin peroxidase isozyme H8 wildtype gene. Strain JBx_137394: *E. coli* DH5α contains pET-21b( +) plasmid cloned with lignin peroxidase isozyme H8 triple mutant with the following mutations: A55R, N156E and H239E.

## Abbreviations

LiP: Lignin peroxidase
VP: Versatile peroxidase
MnP: Manganese peroxidase
LiPH8: Lignin peroxidase isozyme H8
VA: Veratryl alcohol
NIMS: Nanostructure initiator mass spectrometry
AIMD: Ab initio Molecular dynamic
GGE: Guaiacylglycerol-β-guaiacyl ether
MALDI: Matrix-assisted laser desorption
DFT: Density functional theory
SMD: Solvation model based on density
PCM: Polarizable continuum model
COSMO: Conductor-like solvation model
NEB: Nudge Elastic Band
