# Supplementary material for: Experimental and theoretical insights into the effects of pH on catalysis of bond-cleavage by the lignin peroxidase isozyme H8 from Phanerochaete chrysosporium
Source: Biotechnol Biofuels. 2021 Apr 29;14:108. doi: 10.1186/s13068-021-01953-7 (PMC8082889; doi:10.1186/s13068-021-01953-7)
Supplement: Supplementary file 1 — Additional file 1: Figure S1. Relative Gibbs free energy for the formation of protonated intermediates at phenolic OH (blue bar), Cα-OH (green bar) and Cγ-OH (orange bar) positions through a pre-protonation – oxidation route. Gibbs Free energy was normalized to reactants on each reaction step. Figure S2. Relative Gibbs free energy for the formation of protonated intermediates at phenolic OH (blue bar), Cα-OH (green bar) and Cγ-OH (orange bar) positions through a pre-oxidation – protonation route. Gibbs Free energy was normalized to reactants on each reaction step. Figure S3. Snapshots of intermediates from AIMD simulation of the protonated Cα-OH, GGE cationic radical for β-O-4′ ether bond cleavage. Figure S4. Snapshots of intermediates from AIMD simulation of protonated Cγ-OH, GGE cationic radical for β-O-4′ ether bond cleavage. Figure S5. Proposed mechanism for β-O-4′ ether bond cleavage from protonated Cα-OH, GGE cationic radical. Figure S6. Proposed mechanism for β-O-4′ ether bond cleavage from protonated Cγ-OH, GGE cationic radical. [file 13068_2021_1953_MOESM1_ESM.docx]

**Supplementary data**

**Experimental and theoretical insights into the effects of pH on catalysis of bond-cleavage by the lignin peroxidase isozyme H8 from *Phanerochaete chrysosporium***

**Le Thanh Mai Pham^1,2^, Kai Deng^1,2^, Trent R. Northen^1,3^, Steven W. Singer^1,3^, Paul D. Adams^1,3,4^, Blake A. Simmons^1,3^, Kenneth L. Sale^1,2*^**

1. Joint BioEnergy Institute, Emeryville, CA 94608, USA

2. Sandia National Laboratories, Livermore, CA, 94550, USA

3. Lawrence Berkeley National Laboratory, Berkeley, CA 94720, USA

4. University of California, Berkeley, CA 94720

*Correspondence: Kenneth L. Sale ([klsale@lbl.gov](mailto:klsale@lbl.gov))

**Author email**

LTMP: thanhmaipl@lbl.gov

KD: [kdeng@lbl.gov](mailto:kdeng@lbl.gov)

TRN: trnorthen@lbl.gov

SWS: swsinger@lbl.gov

PDA: pdadams@lbl.gov

BAS: basimmons@lbl.gov

KLS: [klsale@lbl.gov](mailto:klsale@lbl.gov)


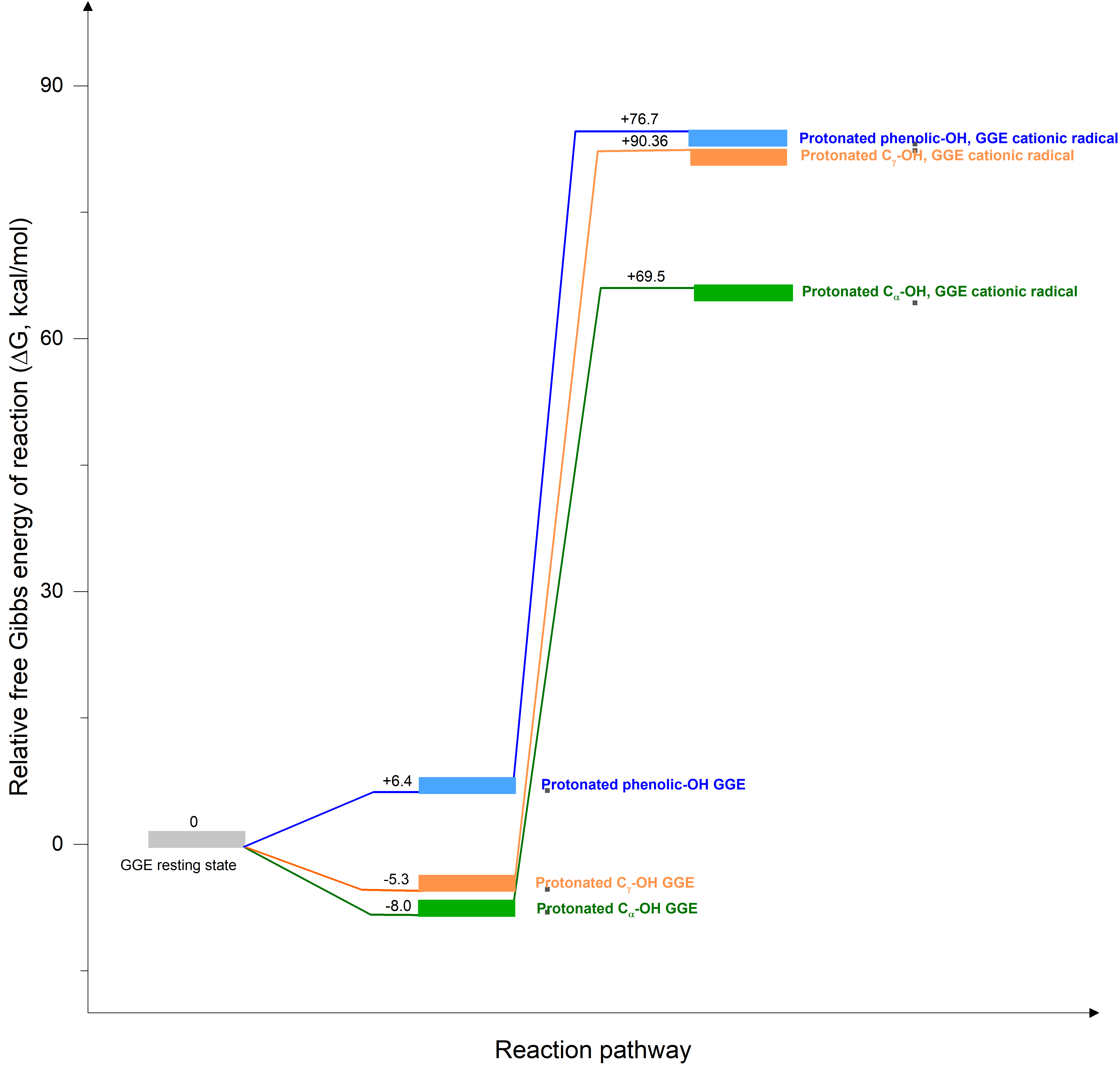


Figure S1. Relative Gibbs free energy for formation of protonated intermediates at phenolic OH (), C_α_-OH () and C_γ_-OH () positions through a pre-protonation – oxidation route. Gibbs Free energy was normalized to reactants on each reaction step.


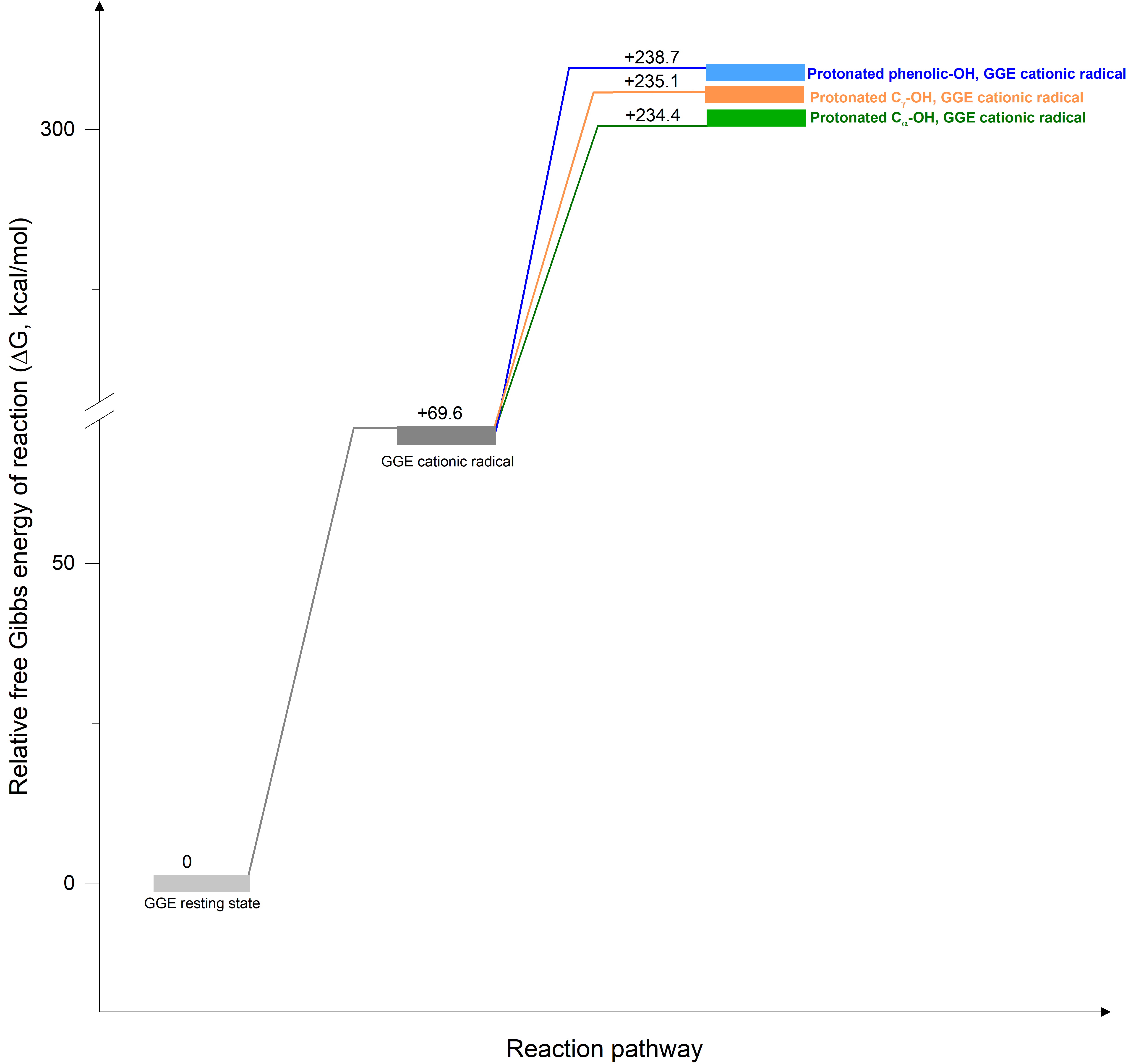


Figure S2. Relative Gibbs free energy for formation of protonated intermediates at phenolic OH (), C_α_-OH () and C_γ_-OH () positions through a pre-oxidation – protonation route. Gibbs Free energy was normalized to reactants on each reaction step.


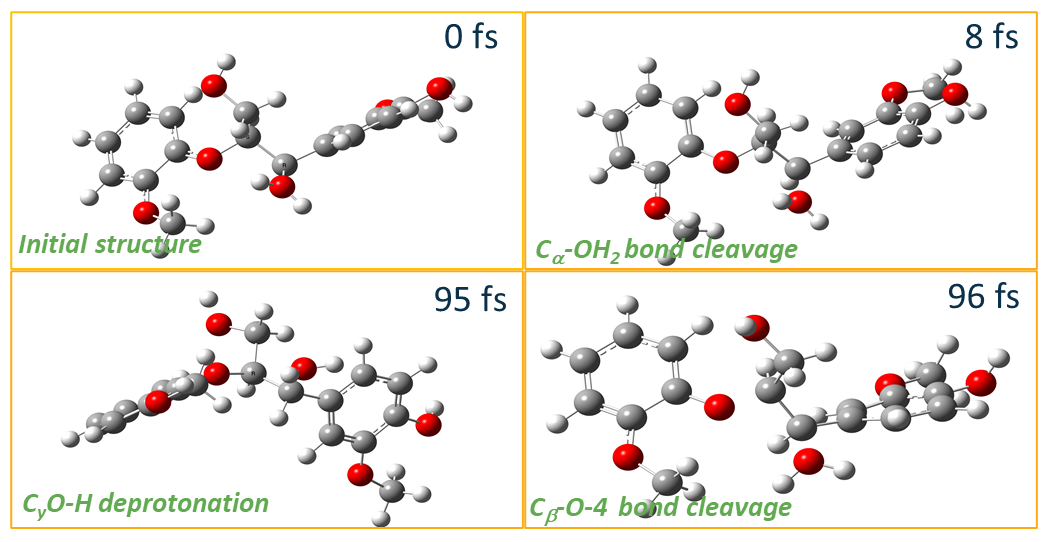


Figure S3. Snapshots of intermediates from AIMD simulation of the protonated C_α_-OH, GGE cationic radical for β-O-4’ ether bond cleavage.


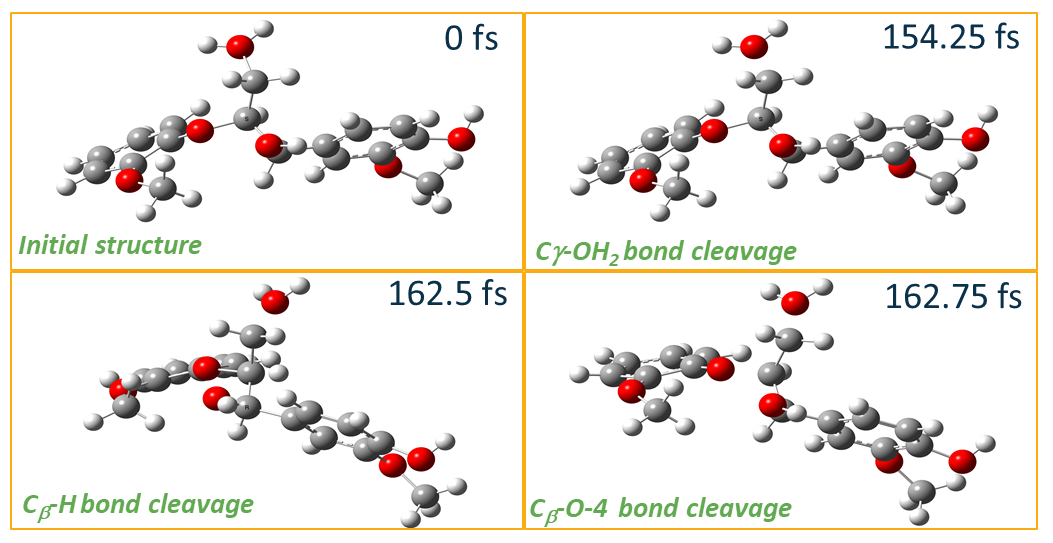


Figure S4. Snapshots of intermediates from AIMD simulation of protonated C_γ_-OH, GGE cationic radical for β-O-4’ ether bond cleavage.

Figure S5. Proposed mechanism for β-O-4’ ether bond cleavage from protonated C_α_-OH, GGE cationic radical.

Figure S6. Proposed mechanism for β-O-4’ ether bond cleavage from protonated C_γ_-OH, GGE cationic radical
